# Evidential deep learning for trustworthy prediction of enzyme commission number

**DOI:** 10.1093/bib/bbad401

**Published:** 2023-11-22

**Authors:** So-Ra Han, Mingyu Park, Sai Kosaraju, JeungMin Lee, Hyun Lee, Jun Hyuck Lee, Tae-Jin Oh, Mingon Kang

**Affiliations:** Department of Life Science and Biochemical Engineering, Sun Moon University, Asan, Republic of Korea; Bio Big Data-based Chungnam Smart Clean Research Leader Training Program, SunMoon University, Asan, Republic of Korea; Bio Big Data-based Chungnam Smart Clean Research Leader Training Program, SunMoon University, Asan, Republic of Korea; Division of Computer Science and Engineering, Sun Moon University, Asan, Republic of Korea; Department of Computer Science, University of Nevada, Las Vegas, NV, USA; Bio Big Data-based Chungnam Smart Clean Research Leader Training Program, SunMoon University, Asan, Republic of Korea; Division of Computer Science and Engineering, Sun Moon University, Asan, Republic of Korea; Bio Big Data-based Chungnam Smart Clean Research Leader Training Program, SunMoon University, Asan, Republic of Korea; Division of Computer Science and Engineering, Sun Moon University, Asan, Republic of Korea; Genome-based BioIT Convergence Institute, Asan, Republic of Korea; Research Unit of Cryogenic Novel Material, Korea Polar Research Institute, Incheon, Republic of Korea; Department of Life Science and Biochemical Engineering, Sun Moon University, Asan, Republic of Korea; Bio Big Data-based Chungnam Smart Clean Research Leader Training Program, SunMoon University, Asan, Republic of Korea; Genome-based BioIT Convergence Institute, Asan, Republic of Korea; Department of Pharmaceutical Engineering and Biotechnology, Sun Moon University, Asan, Republic of Korea; Department of Computer Science, University of Nevada, Las Vegas, NV, USA

**Keywords:** enzyme commission number, evidential deep learning, biologically interpretable deep learning, ECPICK

## Abstract

The rapid growth of uncharacterized enzymes and their functional diversity urge accurate and trustworthy computational functional annotation tools. However, current state-of-the-art models lack trustworthiness on the prediction of the multilabel classification problem with thousands of classes. Here, we demonstrate that a novel evidential deep learning model (named ECPICK) makes trustworthy predictions of enzyme commission (EC) numbers with data-driven domain-relevant evidence, which results in significantly enhanced predictive power and the capability to discover potential new motif sites. ECPICK learns complex sequential patterns of amino acids and their hierarchical structures from 20 million enzyme data. ECPICK identifies significant amino acids that contribute to the prediction without multiple sequence alignment. Our intensive assessment showed not only outstanding enhancement of predictive performance on the largest databases of Uniprot, Protein Data Bank (PDB) and Kyoto Encyclopedia of Genes and Genomes (KEGG), but also a capability to discover new motif sites in microorganisms. ECPICK is a reliable EC number prediction tool to identify protein functions of an increasing number of uncharacterized enzymes.

## INTRODUCTION

Proteins functional annotation is critical for characterizing unknown enzymes to catalyze a wide range of commercial processes, such as pharmaceutical biosynthesis, food production and bioremediation [1]. Functional annotation tools also unveil complex pathways of enzymes involved in biological regulatory processes at the cellular level for diagnosing enzyme-related diseases [2] and prioritize drug targets for efficient biological experiment designs [3]. Enzyme Commission (EC) numbers classify enzyme’s functions, using four digits that hierarchically refers to a class, a subclass, a sub-subclass and a serial number [4, 5].

Trustworthy and accurate computational prediction of EC numbers is urgent to infer new species’ gene properties and biological functions. As of February 2022, 565,928 (manually curated) and 225,013,025 (computationally annotated) numbers of protein sequences have been identified with their EC numbers in the Uniprot database [6]. However, it is reported that only 33% of unknown proteins are matched with such well-characterized enzymes in terms of sequence similarity [7, 8], which indicates that there is still a large number of proteins whose biological functions are not yet known. Furthermore, an increasing number of new species of diverse organisms (e.g. microorganisms) has been reported along with the advance in high-throughput genome sequencing technologies and environmental changes [9].

Recently, machine-learning-based models have been widely proposed for the EC number prediction (§S1 in the supplementary). Current state-of-the-art machine-learning-based models, including MF-EFP [3], DeepEC [10], DEEPred [11], HECNet [12], HDMLF [13] and CLEAN [14], have improved the predictive performance by automatically recognizing class-specific patterns of protein sequences from a large scale of labeled databases. Especially, machine-learning-based models have shown promising performance with unknown enzymes of new species, particularly in microorganisms, compared with protein structured-based (e.g. Cofactor [15], i-tasser suite [16]) and sequence similarity-based approaches (e.g. EFICAz [17], ModEnzA [18], PRIAM [19] and EnzML [20, 21]). However, current machine-learning-based models lack model interpretation and trustworthiness in their predictions. The multilabel classification of >5000 EC numbers potentially cause a large number of false positives [22]. EC number predictions with domain-relevant evidence, such as the presence of essential motif sites in enzymes, can be a solution for trustworthy prediction. For instance, cytochrome P450 (CYP) essentially contains heme as a cofactor functioning as monooxygenases, so the EC predictions with the evidence of heme binding sites would reduce false positives before biological validation. Furthermore, model interpretation identifies amino acids (AAs) that significantly contribute to the prediction, which can potentially discover unknown motif sites [23].

**Figure 1:**
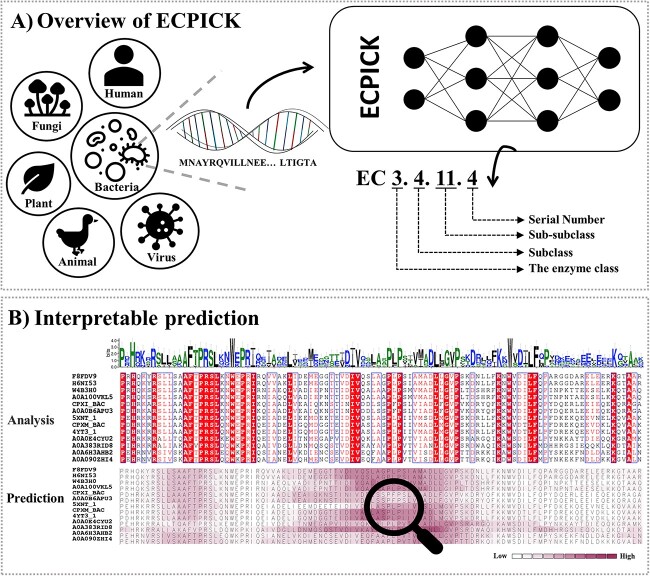
Overview of ECPICK. (A) ECPICK is a biologically interpretable deep learning model that accurately predicts EC numbers from AA sequences. (B) ECPICK identifies significant AAs that contribute to the predictions in a given protein sequence, which may correspond to known motif sites or conserved regions for trustworthy predictions or potential motif sites.

We developed an evidential deep learning model that not only improves the predictive performance of EC numbers in trustworthiness with domain-relevant evidence, but also can identify potential new motif sites (Figure 1). Twenty millions of protein sequence data were used to train our EC number Predictive model, which is based on a biologically Interpretable Convolutional neural network for biological Knowledge discovery (a.k.a. ECPICK). In ECPICK, a protein sequence is fed into convolutional neural network models, and posterior probabilities of around 5000 EC numbers are computed through the model’s convolutional and hierarchical layers for the hierarchical multilabel classification problem. ECPICK identifies significant sequential patterns of AAs associated to the classification of each EC number class, which may correspond to known or potential motif sites. The capability of ECPICK to discover potential motif sites is geared toward biological interpretability, and the AAs identified significant provide qualitative domain-relevant evidence for prediction trustworthiness. We assessed our proposed method, ECPICK, by (i) comparing the predictive performance (F1-scores) with the current state-of-the-art methods using two large protein databases of Swiss-Prot and KEGG in which curated EC numbers are known, (ii) verifying the capabilities to identify potential motif sites for trustworthy prediction and model interpretation and (iii) applying ECPICK to a complete genome of a microorganism, whose characteristics are biologically verified but EC numbers are not completely curated yet. To the best of our knowledge, our proposed model is the first model that provides trustworthy EC number predictions through powerful biological interpretability.

## MATERIAL AND METHODS

### Model design

**Figure 2:**
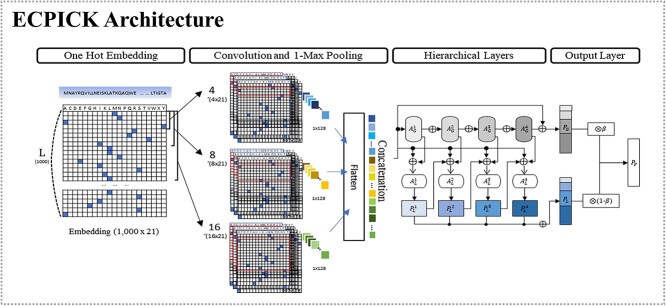
The model architecture of ECPICK. ECPICK consists of the four groups of layers: encoding layer, convolutional layers, hierarchical layers and output layers for the multilabel hierarchical classification problem. The encoding layer converts protein sequence data into a matrix using one-hot-encoding. The convolutional layers include three convolutional layers and 1-max pooling to capture sequential patterns of various lengths and concatenate the representation maps into a vector. The hierarchical layers learn hierarchy of the EC number nomenclature. The output layer finally produces the posterior probability for each EC number with four digits.

The biologically interpretable deep learning model, ECPICK, consists of four groups of layers: (i) encoding layer, (ii) convolutional layers, (iii) hierarchical layers and [iv] output layers (Figure 2). In the encoding layer, a protein sequence is converted to a 1000 × 21 matrix by using one-hot encoding. Each AA is represented by one of 21 alphabet characters, where 20 AAs are conventionally denoted as their AA alphabets, but the AAs of ‘B’, ‘Z’, ‘U’ and ‘O’ are considered as a special codon denoted as ‘X’. The special codons are rarely observed; only 0.1% protein sequences include the special codons in Swiss-Prot. We consider only the first 1000 AAs of a protein sequence, since the sizes of most protein sequences are shorter than 1000 (96% in Swiss-Prot and 98% in PDB) and most important motif sites are observed within the regions. Protein sequences shorter than 1000 are padded by zeros.

The encoded input matrix is introduced to the convolutional layers. The convolutional layers capture AA’s sequential patterns of various sizes. ECPICK considers three convolutional layers with 128 filters of 4 × 21, 8 × 21 and 16 × 21 kernel sizes, where each filter trains sequential AA patterns parallelly in different sizes. The sequential AA patterns in sizes of 4, 8 and 16 produce 997 × 128, 993 × 128 and 985 × 128 activation maps, respectively. Then, 1-max pooling is applied to each activation map, which produces three 1 × 128 vectors. The vectors are concatenated into a vector of 1 × 384. The concatenated vector highlights AA positions where significant sequential patterns associated with the prediction are shown, and the vector is fed into the hierarchical layers.

The hierarchical layers examine the associations between the convolutional features of protein sequences and hierarchically structured labels of EC numbers. The hierarchical layers combine global and local flows of the hierarchy. The global flow (${\mathrm{A}}_{\mathrm{G}}^1-{\mathrm{A}}_{\mathrm{G}}^4$ in Figure 2) solves the hierarchical classification problem as a multilabel classification without hierarchy information, where all leaves of the hierarchical tree are considered an independent class. Let $\mathrm{v}\in{\mathfrak{R}}^{384}$ be the concatenated input vector from the convolutional layer. ${\mathrm{A}}_{\mathrm{G}}^1-{\mathrm{A}}_{\mathrm{G}}^4$ are computed by


(1) \begin{equation*} {\mathrm{A}}_{\mathrm{G}}^1=\phi \left({\mathrm{W}}_{\mathrm{G}}^1\mathrm{v}+{\mathrm{b}}_{\mathrm{G}}^1\right), \end{equation*}

(2) \begin{equation*} {\mathrm{A}}_{\mathrm{G}}^{\mathrm{h}}=\phi \left({\mathrm{W}}_{\mathrm{G}}^{\mathrm{h}}\left({\mathrm{A}}_{\mathrm{G}}^{\mathrm{h}-1}\bigoplus \mathrm{v}\right)+{\mathrm{b}}_{\mathrm{G}}^{\mathrm{h}}\right),2\le h\le 4, \end{equation*}

where ${\mathrm{W}}_{\mathrm{G}}^{\mathrm{i}}\in{\mathfrak{R}}^{s\times 384}$ and ${\mathrm{b}}_{\mathrm{G}}^{\mathrm{i}}\in{\mathfrak{R}}^s$ are weight matrices and bias vectors, respectively ($s$ is a node number of each layer, Data S1 in Supplementary excel data), $\phi$ is an activation function (e.g. ReLU in this study) and $\bigoplus$ is a concatenation operator. The final global prediction is made by


(3) \begin{equation*} {\mathrm{P}}_{\mathrm{G}}=\sigma \left({\mathrm{W}}_{\mathrm{G}}^{\mathrm{H}+1}{\mathrm{A}}_{\mathrm{G}}^{\mathrm{H}}+{\mathrm{b}}_{\mathrm{G}}^{\mathrm{H}+1}\right), \end{equation*}

where $\mathrm{H}$ is a depth of the hierarchical label tree ($\mathrm{H}=4$ in ECPICK), ${\mathrm{W}}_{\mathrm{G}}^{\mathrm{H}+1}\in{\mathfrak{R}}^{C_H\times{A}_G^H}$ and $\sigma$ is a sigmoid activation function to the output layer. On the other hand, the local flow (${\mathrm{A}}_{\mathrm{L}}^1-{\mathrm{A}}_{\mathrm{L}}^4$ and ${\mathrm{P}}_{\mathrm{L}}^1-{\mathrm{P}}_{\mathrm{L}}^4$ in Figure 2) transits the classification information of the class hierarchy. ${\mathrm{A}}_{\mathrm{L}}^{\mathrm{h}},\left(1\le h\le H\right)$ is a latent variable coming from the input vector ($\mathrm{v}$), the global flow representation (${\mathrm{A}}_{\mathrm{G}}^h$) and the previous local flow representation (${\mathrm{A}}_{\mathrm{L}}^{h-1}$). It produces a probability of EC number classes in the $h$-th level (${\mathrm{P}}_{\mathrm{L}}^h$):


(4) \begin{equation*} {\mathrm{A}}_{\mathrm{L}}^1=\phi \left({\mathrm{W}}_{\mathrm{L}}^1\left({\mathrm{A}}_{\mathrm{G}}^1\bigoplus \mathrm{v}\right)+{\mathrm{b}}_{\mathrm{L}}^1\right), \end{equation*}

(5) \begin{equation*} {\mathrm{P}}_{\mathrm{L}}^1=\sigma \left({\mathrm{W}}_{\mathrm{L}}^1{\mathrm{A}}_{\mathrm{L}}^1+{\mathrm{b}}_{\mathrm{L}}^1\right), \end{equation*}

(6) \begin{equation*} {\mathrm{A}}_{\mathrm{L}}^h=\phi \left({\mathrm{W}}_{\mathrm{L}}^{\mathrm{h}}\left({\mathrm{A}}_{\mathrm{G}}^h\bigoplus{\mathrm{A}}_{\mathrm{L}}^{h-1}\bigoplus \mathrm{v}\right)+{\mathrm{b}}_{\mathrm{L}}^{\mathrm{h}}\right),\quad 2 \le h\le 4 \end{equation*}

(7) \begin{equation*} {\mathrm{P}}_{\mathrm{L}}^h=\sigma \left({\mathrm{W}}_{\mathrm{L}}^{\mathrm{h}}{\mathrm{A}}_{\mathrm{L}}^h+{\mathrm{b}}_{\mathrm{L}}^{\mathrm{h}}\right). \end{equation*}

The local flow representations of ${\mathrm{P}}_{\mathrm{L}}^h,1\le h\le 4,$ in the four levels are concatenated into ${\mathrm{P}}_L$:


(8) \begin{equation*} {\mathrm{P}}_L={P}_L^1\bigoplus{P}_L^2\bigoplus{P}_L^3\bigoplus{P}_L^4. \end{equation*}

The final output layer corresponds to the EC number classes in all depths and compute posterior probabilities of the EC numbers given protein sequence, $\Pr \left({\mathrm{C}}_{\mathrm{i}}|\mathrm{x}\right)$, by combining the scores of global and local flows with weights ($\beta$):


(9) \begin{equation*} {P}_F=\beta{\mathrm{P}}_{\mathrm{G}}+\left(1-\beta \right){\mathrm{P}}_{\mathrm{L}}, \end{equation*}

where $\beta \in \left[0,1\right]$ is the parameter that regularizes the trade-off the local and global flows. The details of the ECPICK architecture are elucidated in Data S1 in Supplementary excel data.

ECPICK minimizes the sum of the local and global loss functions for hierarchical multilabel classification with the objective function:


(10) \begin{equation*} \mathrm{L}=\ell \left({\mathrm{P}}_{\mathrm{G}},\mathrm{Y}\right)+\ell \left({\mathrm{P}}_{\mathrm{L}},\mathrm{Y}\right), \end{equation*}

where $\mathrm{Y}$ is the binary class vector containing the one-hot encoded EC number classes in all depths for the hierarchical multilabel classification problem. We consider the binary cross-entropy for the loss functions locally and globally:


(11) \begin{equation*} \ell \left(\hat{\mathrm{Y}},\mathrm{Y}\right)=-\frac{1}{N}\sum_{i=1}^N\sum_{j=1}^{\mid C\mid}\left({Y}_{ij}\log \left(\hat{Y_{ij}}\right)+\left(1-{Y}_{ij}\right)\log \left(1-\hat{Y_{ij}}\right)\right), \end{equation*}

where $\hat{\mathrm{Y}}$ is posterior probabilities of EC numbers, $\mathrm{N}$ is a sample size and $\mathrm{C}$ is class numbers. For the final EC number predictions, we consider only the posterior probabilities of the four-digit EC numbers on the fourth level.

We improve the model’s robustness by adopting a simple ensemble approach. We trained the model 10 times with random initiations on the model parameters and obtained the 10 optimal models of ECPICK, including posterior probabilities of EC number prediction (Table S1). The 10 posterior probabilities of EC numbers are averaged for the final prediction. More than 10 models did not significantly improve predictive performance. Datasets (§S2), ablation study (§S3), hyper-parameter tuning (§S4) and optimization of threshold for the multilabel classification (§S5) are detailed in the supplementary.

### Computing importance scores for trustworthy predictions and identifying potential motif sites

Trustworthiness of machine learning models is critical when facilitating AI systems for high-stakes decisions [24]. Trustworthy machine learning clarifies the process of how a model predicts while often providing feature-based evidence (e.g. essential motif sites in a given enzyme). ECPICK produces trustworthy EC number predictions by identifying significant AAs to the prediction as biological supports, which may correspond to known motif sites of a given protein. Furthermore, the capability of ECPICK to identify significant AAs in a given protein sequence allows one to discover unknown motif sites or domains, which can be new biological knowledge. ECPICK computes importance scores on each AA to quantitatively measure the significance of sequential patterns over the neighboring AAs that contribute to the predictions (§S6 in the supplementary). ECPICK also visualizes them by matching them with known motif sites, conserved sequences or domains as qualitative evidence for the EC number classification.

## RESULTS AND DISCUSSION

### ECPICK outperforms benchmark methods when predicting enzyme EC numbers in Swiss-Prot and KEGG

We assessed the predictive performance of our proposed model, ECPICK, by comparing it with current state-of-the-art methods using curated protein sequence data in the Swiss-Prot and KEGG databases. In the Swiss-Prot database, we considered curated enzyme sequence data registered since August 2020, which we did not use to train our model. In the KEGG database, we considered the complete genome sequence data of the three species of bacteria, whose experiments or biological functions are well known and have been widely used as genome assembly references. Swiss-Prot consists of individually curated enzymes of various organisms, whereas KEGG includes annotated enzyme genes in diverse organisms’ complete genome sequences to uncover cellular and organism-level functions by reconstructing specific metabolic pathways [25].

In the Swiss-Prot database, we examined 858 new samples among the newly added 2567 protein sequences between August 2020 and April 2021, where we excluded: (i) 156 protein sequences whose AA length was <10 and (ii) 1553 protein sequences whose EC numbers were newly registered, so they were not included in the final ECPICK model. For the evaluation, we computed micro-averaged precision/recall/F1-scores, execution time and prediction numbers, due to extremely imbalanced data and validation with only a small number of EC numbers (Figure S2). For the benchmark models, we included DeepEC, ECPred, EFICAz2.5 and DTECTv2, while excluding unavailable models due to service termination, invalid service and version conflict. The final model of ECPICK was trained with the optimal hyper-parameters and datasets from Swiss-Prot, TrEMBL and PDB, published before August 2020.

**Figure 3:**
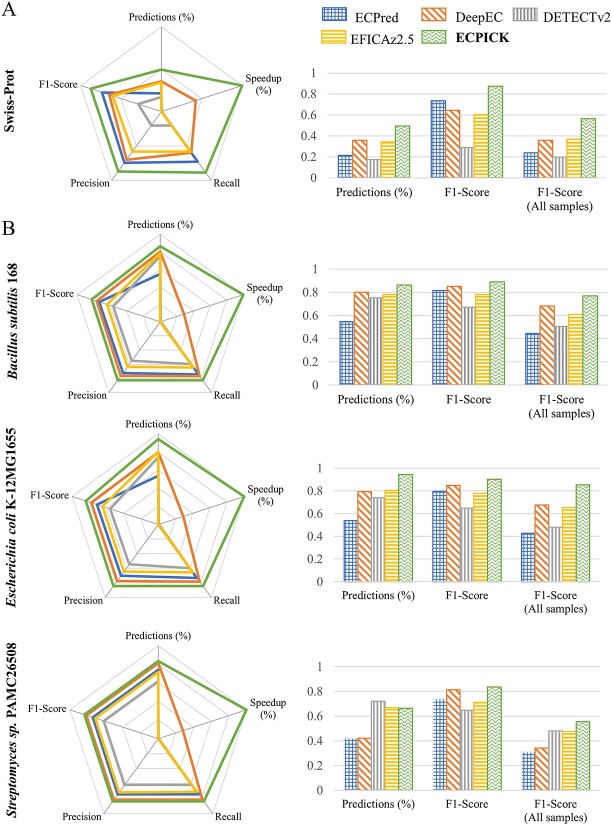
Performance comparison using Swiss-Prot (A) and KEGG databases (B). (A) ECPICK’s predictive performance of ECPICK was evaluated using individually curated enzymes in Swiss-Prot and annotated enzyme genes from the complete genome sequences of the three bacteria. (B) B*. B. subtilis* strain 168, *E. coli* strain K-12 MG1655 and *Streptomyces* sp. strain PAMC 26508. The performance of ECPICK was compared with ECPred, DeepEC, DETECTv2 and EFICAz2.5, where the radar and bar charts are linearly scaled between 0 and 1. Precision, recall, F1-score, numbers of prediction and speed up (%) are illustrated, and actual values are available in Tables S2 and S3.

ECPICK showed superior F1-scores to the benchmarks while predicting the most EC numbers in the experiments (Figure 3A and Table S2). Given the optimal threshold (FDR < 0.05), ECPICK identified EC numbers of 407 enzymes out of 858, of which 360 EC number predictions were correct. The micro-averaged F1-score of ECPICK was 0.8759 on the 407 predictions (values without parenthesis in Table S2), and the F1-score on all the given samples, including samples that did not predict due to the threshold, was 0.5660 (in parenthesis in Table S2). The second-best model was ECPred, which showed F1-scores of 0.7349 and 0.2387 with 184 predictions and the entire samples, respectively. ECPICK produced 137% and 57% improved predictive performance on 121% and 32% more enzymes than ECPred and DeepEC, respectively. Interestingly, ECPICK was the only model that predicted the EC 7 category. In 2018, the EC reorganized the nomenclature by adding the EC 7 category (the first level) to elucidate the enzyme group that catalyzes the movement of ions or molecules across membranes or their separation within membranes. ECPICK correctly predicted 20 enzymes of EC 7, but none of the other benchmark models identified enzymes of EC 7. All the results are available in Data S2 in Supplementary excel data.

Among the 47 enzymes that ECPICK misclassified, 39 enzymes were correctly classified up to the third level but misclassified only on the fourth level (Data S3 in Supplementary excel data). EC numbers up to the third level refer to biological functions, whereas the fourth level represents reaction to substrate. Thus, the discrepancy in the fourth level in the prediction of ECPICK may present a potential reaction to another substrate rather than misclassification. Among the remaining eight misclassified enzymes, the following three predictions were actually correct or legitimate. (i) ECPICK predicted EC 7.1.1.2 for the protein sequence of *Q9VZU4 *(access number), known as EC 1.6.5.3. EC 1.6.5.3 was replaced with EC 7.1.1.2 in 2018 [26]. Thus, ECPICK’s prediction was correct, but the ground truth was not up-to-date in the database. (ii) ECPICK predicted to EC 1.5.1.5 for the protein sequence of *P11586*, known as EC 6.3.4.3. The enzyme, *P11586*, is a multifunctional enzyme of methylenetetrahydrofolate dehydrogenase (NADP^+^) (i.e. EC 1.5.1.5), methyltetrahydrofolate cyclohydrolase (i.e. EC 3.5.4.9) activity or formate-tetrahydrofolate ligase (i.e. EC 6.3.4.3) in eukaryotes [27]. Hence, the ECPICK’s prediction of EC 1.5.1.5 refers to one of the multi-biological functions of the enzyme. (iii) ECPICK predicted to EC 2.7.12.2 for the protein sequence of *G4NEB8*, known as EC 2.7.11.24. EC 2.7.12.2 and EC 2.7.11.24 have a functional dependency on each other, where EC 2.7.12.2 is required to activate EC 2.7.11.24. In Uniprot, the function of *G4NEB8* is described as mitogen-activated protein kinase kinase, but is registered as EC 2.7.11.24 in the database. According to the functions assigned to the EC number, EC 2.7.11.24 corresponds to mitogen-activated protein kinase, whereas EC 2.7.12.2 is defined as a mitogen-activated protein kinase kinase. Hence, *G4NEB8* is expected to be EC 2.7.12.2, rather than EC 2.7.11.24. Consequently, ECPICK produced a biologically acceptable predictive performance of 99% throughout all its predictions.

ECPICK showed the most efficiency among the benchmarks. It performed an EC number prediction for the 858 samples in 11 s, which is a 57.6% improvement from the second best, DeepEC, and a 99.3% improvement from the third best, DETECTv2. All experiments were performed on the same workstation with CPU of Intel i9-10940x, 256GB RAM, GPU of RTX 2080 Ti. ECPICK required 1 GB memory on GPU through the data streaming technology and took 8 h per epoch for training.

Furthermore, we verified ECPICK’s predictive performance using curated complete genome sequence data in KEGG, which is one of the largest molecular databases. KEGG annotated EC numbers of the protein sequences in complete genomes using KEGG mapping [25, 28], which were considered ground truth in this experiment. We examined the reference genome assembly of species of three bacteria: (i) *Bacillus subtilis* 168, (ii) *Escherichia coli* K-12 MG1655 and (iii) *Streptomyces* sp. PAMC26508 in KEGG. Strain *B. subtilis* 168 and *E. coli* K-12 MG1655 are well-known references for various genomic analysis [29, 30]. Whereas, *S.* sp. PAMC 26508 is the first isolated actinomycetes from Antarctic lichen [31], where various computational tools have characterized their EC numbers in KEGG. The KEGG database contained 1057, 1283 and 1398 annotated enzymes in the three-bacteria genome information, respectively. ECPICK outperformed the benchmark models in most evaluation metrics. It also showed the highest micro-averaged F1-scores of 0.89, 0.90 and 0.83 in the three species, respectively, in the predicted samples, while identifying most EC number predictions, 913 out of 1057 (86%), 1211 out of 1283 (94%) and 922 out of 1389 (66%). Including all samples that ECPICK could not predict due to thresholding, ECPICK’s F1-scores were 0.77, 0.85 and 0.55, respectively, which showed 12%, 26% and 15% improvements against the second best in the three species (Figure 3B and Table S3).

### Evidential deep learning model, ECPICK, provides trustworthy predictions and identifies potential motif sites

We verified ECPICK’s domain-relevant evidence computed by the importance scores, using well-reported motif sites on CYPs enzyme sequences. CYPs are monooxygenases that catalyze the incorporation of a single oxygen atom into a substrate [32, 33], existing in most organisms, including bacteria, fungi, plants, insects and mammals. In particular, we considered the CYP106A2 family sequences (i.e. EC 1.14.15.8) in bacteria and CYP7B1 family sequences (i.e. classified as EC 1.14.14.26 or 1.14.14.29) in mice, rats and humans for the assessment. CYP106A2 and CYP7B1 enzymes share similar biological functions with each other but in different organisms. The bacterial CYP106A2 group plays an essential role in attaching a hydroxyl group to a steroid structure within the bacterial CYP enzyme groups. On the other hand, CYP7B1 is a CYP group involved in the metabolism of endogenous oxysterols and steroid hormones as human CYP groups, including neurosteroids in the eukaryotic cell. The CYP group function of prominent hydroxyl groups to carbon positions of the substrate and the binding characteristics have been widely used in the pharmaceutical industry and clinical/disease-related medicines.

**Figure 4:**
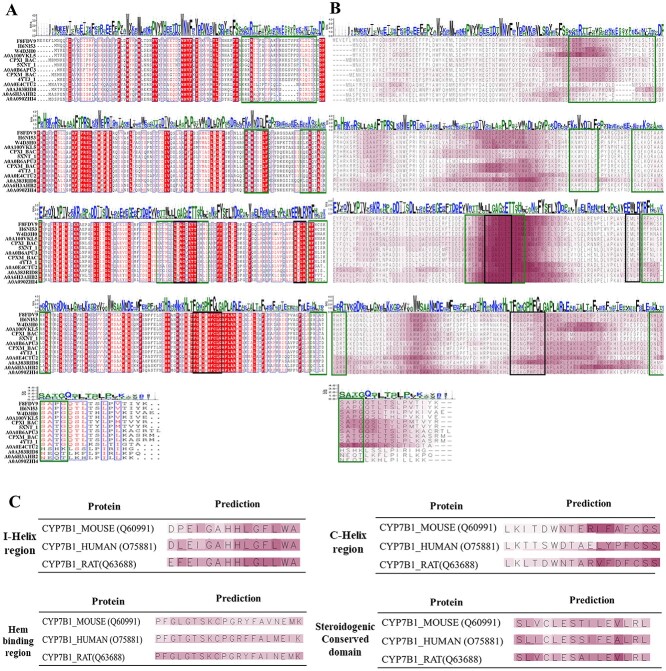
ECPICK identifies significant AAs contributing to the prediction for trustworthy EC number predictions and can discover potential motif sites. (A) It illustrates conserved sequences, motif sites (dotted lines in black) and substrate recognition sites (solid lines in green) of the CYP106A2 family. (B) The figure shows the importance scores of the enzymes, which are well aligned with the known motif sites, substrate recognition sites and conserved domains in the left side (A). The importance scores are also well aligned over the multiple enzymes in the same EC category after multiple sequence alignment. (C) The four motif sites in the protein sequences of the CYP7B1 family are visualized to compare with the importance scores of ECPICK. The importance scores were well aligned with most known motif sites and conserved sequences.

For the bacterial CYP106A2 enzymes, we investigated 13 protein sequences, including *5XNT* and *4YT3* in PDB [34, 35] and additional 11 protein sequences in Swiss-Prot showing >90% sequence similarity with *5XNT* and *4YT3*. ECPICK correctly classified all of them as EC 1.14.15.8. For the trustworthy prediction with domain-relevant evidence, ECPICK computed the importance scores of the protein sequences. The importance scores are visualized with multiple sequence alignment by Clustal Omega [36]. Figure 4A illustrates conserved sequences colored in red, motif sites (oxygen binding, EXXR and hem binding domain in order; dotted lines in black), and substrate recognition sites (solid lines in green; SRS 1–6) of the CYP106A2 family on the left side. ECPICK’s importance scores are visualized on the right (Figure 4B), where high (or low) scores of AA are colored in red (or white). The distribution of the importance scores is well aligned over the 13 enzymes, which implies that the importance scores are reproducible. Note that the computation of the importance scores was conducted individually on each protein sequence before the multiple sequence alignment. Furthermore, the importance scores of ECPICK were well matched with (i) most conserved sequences, (ii) substrate recognition sites of SRS 1, 4–6 and (iii) the essential motif sites of oxygen-binding motif and heme-binding domain that determine the first and second EC levels (e.g. 1.14). The EXXR motif was not identified in ECPICK. It is because EXXR may not be discriminative, specifically when only classifying the corresponding EC number.

We also examined three protein sequences of CYP7B1 family in mice, rats and humans in Swiss-Prot to confirm the reproducibility of the importance scores in the other organisms. ECPICK correctly classified them as EC 1.14.14.26 and highlighted the essential motif sites of the CYP enzyme family, including the oxygen-binding motif, EXXR motif of I-heliox and C-heliox region, heme-binding domain and Steroidogenic conserved domain (Figure 4C). The reproduced high importance scores were shown in the three essential motif sites on the CYP7B1 family as well as the CYP106A2 family. The identified sequential patterns were well aligned with conserved domains or existing/potential motif sites, without high-cost and time-consuming computational processes for sequence similarity and secondary structure comparison. It is worth noting that ECPICK predicted the three enzymes correctly, although the sequence size of the rat CYP7B1 is 18% shorter than the others, which may cause misclassification with similarity-based models. The sizes of the AA sequences of the CYP7B1 family in mice, rats and humans were 507, 505 and 414, respectively.

### ECPICK accurately annotates high-throughput complete genome for metabolic pathway analyses

We evaluated ECPICK as a tool to accurately annotate EC numbers of enzymes from a complete genome from scratch and to specify enzyme families involved in a metabolic pathway of interest. We applied ECPICK to a high-throughput complete genome sequence of a microorganism to characterize pathways, as it is a typical practice of genome study. We considered *S.* sp. PAMC26508 in Actinomycetes [37, 38], which produces secondary metabolite products with diverse genes, expecting various biological functions, such as antibacterial activity [39]. Although the strain’s functions have been widely reported by several computational tools (e.g. in KEGG), their biological functions have not yet been fully explored by biological experiments, and most of their EC numbers are not yet known.

**Figure 5:**
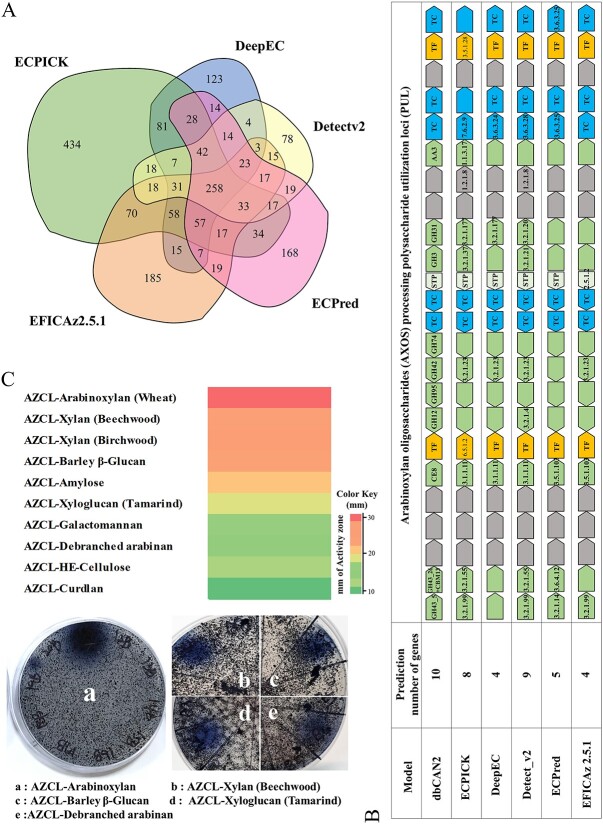
ECPICK accurately annotated the complete genome sequence of *Streptomyces* sp. PAMC 26508. (A) A Venn diagram comparison of ECPICK’s EC number predictions with the benchmark models. A total of 63% of the predictions were also confirmed with at least one other model, while only ECPICK identified 64 of EC 7’s enzymes. (B) The schematic representation of AXOS processing PUL gene cluster and EC number predictions. CAZymes genes, TC, transcription factor (TF) and non-signature genes (between signature genes) are presented. (C) We confirmed CAZyme activity of *Streptomyces* sp PAMC 26508 from AZCL assay. CAZyme activity screening in 10 AZCL substrates and AZCL plate diffusion assay (a. Arabinoxylan; b. Xylan (Beechwood); c. Glucan; d. Xyloglucan and e. Arabinan).

We downloaded the complete genome sequence at the NCBI database. The GenBank accession numbers were CP003990 for chromosome and CP003991 for plasmid. We extracted 6953 protein sequences using Rapid Annotation using Subsystem Technology (RAST) [40, 41] from the complete genome sequence of strain *S.* sp. PAMC26508. Then, we predicted EC numbers of the 6953 protein sequences using ECPICK. Among the 6953 protein sequences, ECPICK identified EC numbers of 1201 enzymes with the threshold of FDR < 0.05 (Data S4 in Supplementary excel data), where ECPICK identified more than two EC numbers on 40 enzymes (i.e. multifunctional enzymes). To verify the reliability of ECPICK’s prediction, we counted how many ECPICK’s predictions overlapped with the other benchmark models, as there are no ground truths in this experiment. At least one of the four benchmark models also predicted the same EC numbers as ~63% of ECPICK’s number predictions (767 out of the 1201 enzymes) (Figure 5A).

ECPICK identified new EC numbers from 434 enzymes that the other benchmarks failed to do. Among the new EC number predictions of the 434 enzymes, ECPICK annotated 64 enzymes as category EC 7 (14.7%; 64 out of the 434 predictions), whereas none of the other benchmark models predicted EC 7. The function of the translocase enzyme family (known as EC 7) has been reported as the most common secretion system in gram-positive bacteria [42]. *S.* sp PAMC26508 is a gram-positive bacterium, which is expected to include enzymes of EC 7. Specifically, ECPICK identified EC 7.1.1.8 [43] and EC 7.1.1.9 [37] from the given enzymes, which were originally assigned EC 1.10.2.2 and EC 1.9.3.1 by RAST, respectively. EC 1.10.2.2 and EC 1.9.3.1 are newly referred to EC 7.1.1.8 and EC 7.1.1.9 in the new nomenclature, respectively. Moreover, among the 64 enzymes of EC 7, 18 protein sequences were predicted as EC 7.4.2.10. EC 7.4.2.10 is classified as prokaryotic ATP-binding cassette (ABC) type transporter, or it is characterized by the presence of two similar ATP-binding domains/proteins and two integral membrane domains/proteins. RAST reported the 18 enzymes as ABC transporters but did not assign relevant EC numbers. Similarly, the other benchmark models failed to predict the EC numbers or predicted the deprecated EC numbers that EC 7 replaced.

Next, we characterized a metabolomic pathway from carbohydrate-active enzymes (CAZymes) of the strain *S.* sp. PAMC 26508 genome sequence to unveil hidden metabolomic functions. We identified 342 CAZyme-related genes using dbCAN tool [44] in the complete genome sequence. Then, we further refined the CAZyme genes by performing Signal P Ver. 4.0 [45] (e-value <1e-15, coverage >0.35), DIAMOND [46] (e-value <1e-102) and Hotpep [47] (frequency > 2.6, hits >6) (Data S5 in Supplementary excel data). Finally, we confirmed 185 protein sequences as CAZymes. ECPICK classified EC numbers of the 185 CAZymes and verified that the predicted EC numbers are matched with the known bacterial functions using KEGG and NCBI BLAST. It is worth noting that Lytic Polysaccharide Mono Oxygenases (LPMOs) were identified by ECPICK as EC 1.14.99.54. LPMO is a traditional hydrolase that oxidizes glycosidic bonds of the recalcitrant polysaccharides [48]. The LPMO families are classified as auxiliary activities and grouped into eight families (AA 9–11, AA 13–17), and are highly abundant in the fungal and streptomyces species genome. However, the interactions of substrates with LPMO have seldom been explored; there are only three biologically reviewed protein sequences in Swiss-Prot and 23 unreviewed sequences in TrEMBL. Nonetheless, ECPICK predicted two enzymes as EC 1.14.99.54, which refers to the degradation of cellooligisaccharides, or chitin. We also confirmed the high similarity (>60%) of the AA sequences between the two enzymes and the known LPMO family. Note that RAST and dbCAN2 predicted them as cellulose-binding enzyme and chitinase (EC 3.2.1.14) and AA10 + CBM2, respectively.

We further conducted a pathway-based analysis, focusing on Arabinoxylan oligosaccharides (AXOS) processing polysaccharide utilization loci (PUL), in the strain PAMC26508 through the gene cluster analysis to determine the strain’s functional roles. The gene cluster analysis identified 24 putative genes associated with the AXOS PUL, and dbCAN2 identified 10 CAZyme families, including eight glycoside hydrolases (GH) (e.g. GH43_5, GH43_26, GH12, GH95, GH42, GH74, GH3 and GH31), carbohydrate esterase family 8 (CE8) and auxiliary activities family 3 (AA3) (Figure 5B). Among the 24 genes, ECPICK predicted EC numbers of the following eight encoding genes: Arabinan endo-1,5-alpha-L-arabinosidase (EC3.2.1.99; fig|1265601.25.peg.5894), Alpha-L-arabinofuranosidase (EC3.2.1.55; fig|1265601.25.peg.5895), Pectinesterase (EC3.1.1.11; fig|1265601.25.peg.5899), Exo-(1,4)-beta-D-galactanase (EC 3.2.1.23; fig|1265601.25.peg.5901), Alpha-D-xyloside xylohydrolase (EC 3.2.1.177; fig|1265601.25.peg.5904), Betaine-aldehydedehydrogenase (EC1.2.1.8; fig|1265601.25.peg.5911), Choline oxidase (EC1.1.3.17; fig|1265601.25.peg.5912) and ABC-type quaternary amine transporter (EC7.6.2.9; fig|1265601.25.peg.5913). On the other hand, DeepEC, DETECTv2, ECPred and EFICAz2.5 predicted four, nine, five and four numbers of genes with their EC numbers, respectively (Figure 5B).

We analyzed the predictive results of the 24 genes in comparison with the benchmark models. (i) ECPICK, DETECTv2 and EFICAz 2.5 classified GH43_5 as EC 3.2.1.99. CAZy referred GH43_5 to EC 3.2.1.99 (endo-α-1,5-L-arabinanase), and BLAST annotated GH43_5 as Arabinanase family with a high similarity (>60%) on Swiss-Prot and PDB. Whereas ECPred predicted it as EC 3.2.1.14 (Chitinase; the enzyme has the activity to chitin and randomly cleaves glycosidic linkage). (ii) GH43_26 is known as the enzyme that responds to Arabinan, such as α-L-arabinofuranosidase (EC 3.2.1.55) in CAZy. ECPICK and DETECTv2 annotated it as EC 3.2.1.55. ECPred predicted it as EC 3.6.4.12 (related enzyme to DNA helicase, not the CAZyme family). (iii) CE8 is reported as an enzyme that responds to pectin, such as pectin methylesterase (3.1.1.11) in CAZy. ECPICK, DeepEC and DETECTv2 annotated it as EC 3.1.1.11, whereas ECPred and EFICAz2.5 predicted it as EC 3.5.1.103, which refers to an enzyme related to mycothiol biosynthesis pathway. (iv) GH42 is an enzyme that responds to lactose or galactose, such as β-galactosidase (EC 3.2.1.23) in CAZy. All models annotated it as the same EC number (EC 3.2.1. 23). [5] GH3 and GH31 contain several enzymes with various EC numbers in CAZy. ECPICK and DETECTv2 predicted GH3 as EC 3.2.1.37 (xylan 1,4-β-xylosidase) and EC 3.2.1.21 (β-glucosidase), respectively. Either of the EC numbers corresponds to GH3 family. [6] ECPICK and DeepEC classified GH31 as EC 3.2.1.177 (α-xylosidase), whereas DETECTv2 predicted it as EC 3.2.1.20 (α-glucosidase). Both of them correspond to the GH31 family. [7] For AA3, only ECPICK classified it as 1.1.3.17, which refers to Choline oxidase. [8] For transporters classification (TC) after AA3, ECPICK classified it as EC 7.6.2.9, referring to an ABC-type quaternary amine transporter. The other models predicted it as 3.6.3.-, which was replaced with EC 7.

Then, we confirmed the bacterial functions of the AXOS degradation pathway by biological experiments with 10 substrates to assess the ECPICK’s prediction of the GH families in CAZyme (Figure 5C). We cultured the strain for 7 days at 15°C and investigated the enzyme activity profiles of 10 Azo-dyed and azurine cross-linked (AZCL) substrates to screen the GH activities (§S7 in the supplementary). Overall, the strain PAMC26508 showed a high hemicellulose degrading activity, especially on AZCL-xylan, AZCL-arabinose and AZCL-beta glucan containing polysaccharides. The strain reacted strongly on xylan but less so on xyloglucan among xylose-containing polysaccharides. That indicates that the strain has a degradation activity to arabinose-containing polysaccharides, and arabinose, xylose, and glucuronate obtained by hemicellulose degradation. Thus, the experiment implies that the strain PAMC26508 involves activities related to arabinoxylan, xylan, cellulose and glucan.

## CONCLUSION

In this paper, we present a novel evidential deep learning model that offers trustworthy predictions for the multilabel classification problem of EC number prediction. Our proposed model, ECPICK, learns complex sequential and hierarchical patterns of protein sequences on thousands of EC number classes from the 20 million’s protein sequences in the largest datasets of Swiss-Prot, TrEMBL and PDB and produces posterior probabilities of EC numbers. The significant improvement of the predictive performance over the statue-of-the-art methods was assessed through multiple intensive experiments. ECPICK computes importance scores on each AA in a given protein sequence, which may correspond to known motif sites or conserved regions for trustworthy prediction, or potentially new motif sites. The importance scores in ECPICK allow one to enjoy the advantage of the protein structure-based approaches, which can effectively identify the most similar folds and functional sites without high-quality protein structure databases or time-consuming computations of multiple sequence alignment. A web service of the model and open-source codes are publicly available at http://ecpick.dataxlab.org and https://github.com/datax-lab/ECPICK, respectively.

Minor variations in sequences could result in functional divergence, leading an enzyme to perform more than one enzymatic function with varying degrees, e.g. multifunctional enzymes or enzyme promiscuity. ECPICK is a probabilistic model that computes a posterior probability on each EC number to assign multiple EC number labels on a given protein sequence with a user defined threshold. The optimal thresholds of the false discovery rates of 5% and 1% are considered in this study for reliable predictions and reducing false positives. Lower thresholding would assign more potential EC numbers on the predictions to broaden understanding of enzyme functions, especially for unknown protein functions and enzyme promiscuity.

To the best of our knowledge, ECPICK is the first evidential deep learning, which is a potential solution for trustworthy prediction of EC numbers and new biological discoveries. However, a quantitative evaluation strategy of the importance score computation is still lacking. Well-known structural information of proteins in the literature or databases (e.g. motif sites, binding sites and domains in Unit-Prot) can be leveraged to match with the AAs identified by the model, as true positive, for the evaluation as a future work. In this study, we evaluated high-scored AAs by (i) comparing the distributions of the importance scores with other protein sequences in the same EC category and (ii) matching with known motif sites or conserved sites. However, it is challenging to define negative AAs in confusion matrices to compute the overall performance.

With the explosion of protein sequences in databases, it is desirable to explore the feasibility of selectively classifying newly discovered enzyme sequences into their respective enzyme classes by means of an automated method. This is important because knowing which family or subfamily an enzyme belongs to may help deduce its catalytic mechanism and specificity, giving clues to the relevant biological function.

**Key Points**

- Our novel tool, ECPICK, offers trustworthy enzyme number predictions with data-driven domain relevant evidence for automatic protein functional annotation.
- Intensive experiments with Swiss-Prot and PDB database shows that ECPICK outperformed the current benchmark models in terms of F1-scores and efficiency.
- ECPICK identifies potential and existing motif sites for reliable EC number prediction and new motif site discovery, without complex post hoc processes, such as multiple sequence alignment.
- A Python package, open-source codes and a web site with graphical interfaces are publicly available, so that anyone can easily utilize the proposed method.

## Supplementary Material

Supplementary_Figures_bbad401

Supplementary_Table_S1_bbad401

Supplementary_Table_S2_bbad401

Supplementary_Table_S3_bbad401

Supplementary_data_bbad401

BB_ECPICK_Supplementary_Final_bbad401

## Data Availability

All data are available at our project web page: http://ecpick.dataxlab.org. All other relevant data are available from the authors upon request. Source data are provided with this paper. The source code to train the ECPICK model and the optimal parameters are available for research and non-commercial use at our github page: https://github.com/datax-lab/ECPICK. A web service of the model is available at http://ecpick.dataxlab.org.
