# Supplementary material for: Evidential deep learning for trustworthy prediction of enzyme commission number
Source: Brief Bioinform. 2023 Nov 22;25(1):bbad401. doi: 10.1093/bib/bbad401 (PMC10664415; doi:10.1093/bib/bbad401)

Fig. S1. Thresholding for the final decision with FDR

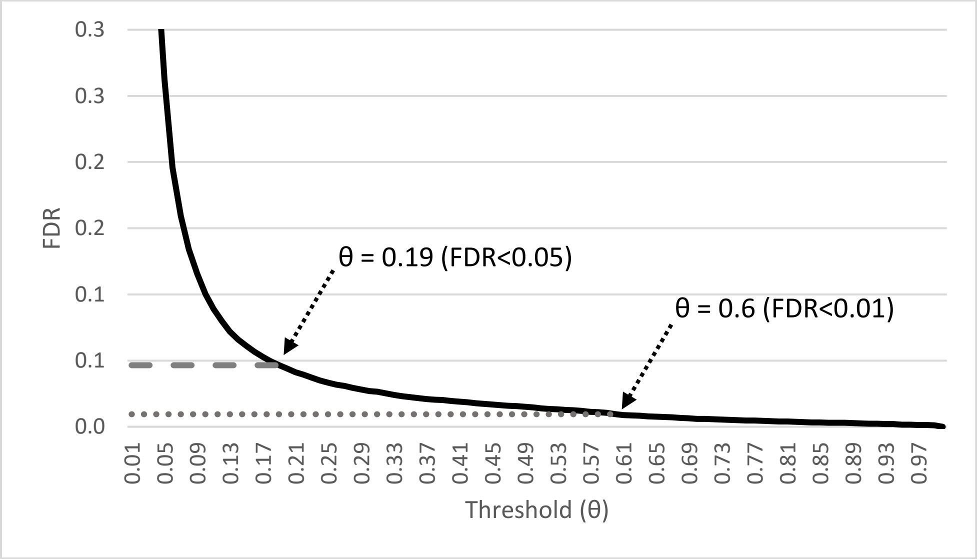

Fig. S2. The sample numbers of the EC classes in the newly registered 858 enzymes. 32 out of 858 EC numbers include more than 5 samples in the test.

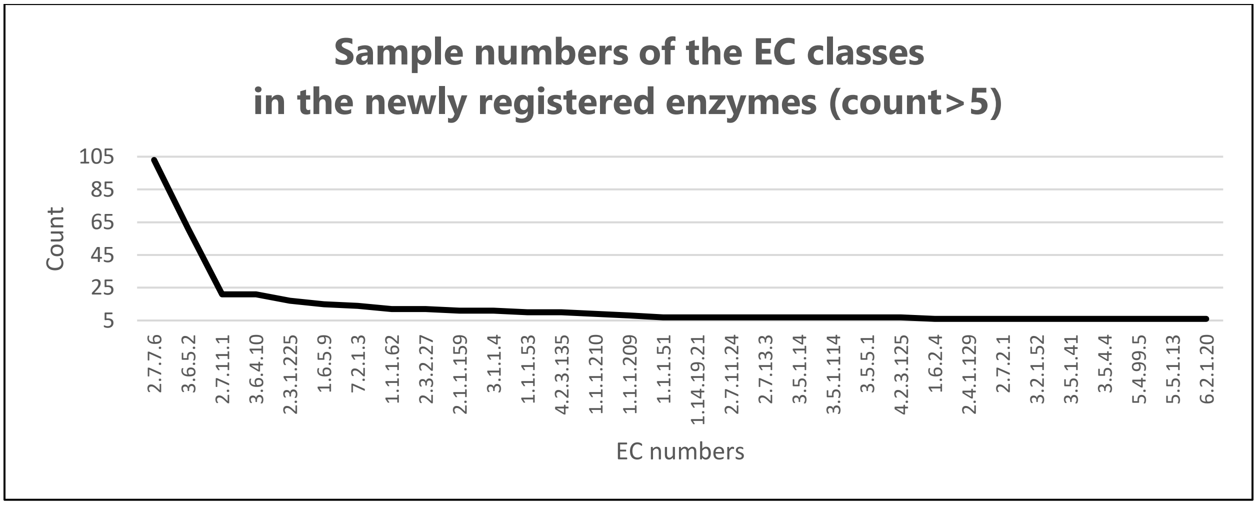

Supplement: Supplementary_Figures_bbad401 [file supplementary_figures_bbad401.pdf]
