# Supplementary material for: Evidential deep learning for trustworthy prediction of enzyme commission number
Source: Brief Bioinform. 2023 Nov 22;25(1):bbad401. doi: 10.1093/bib/bbad401 (PMC10664415; doi:10.1093/bib/bbad401)
Supplement: Supplementary_Table_S1_bbad401 [file supplementary_table_s1_bbad401.pdf]

Table S1. Enhancement of macro F1-scores over the number of ECPICK models for ensemble learning.

No. of models for ensemble	Top 1	Top 2	Top 5
1	0.798792	0.874647	0.911256
2	0.814838	0.890468	0.925237
3	0.818604	0.896750	0.930437
4	0.825190	0.899796	0.934061
5	0.826110	0.901823	0.935470
6	0.827634	0.902564	0.937371
7	0.829292	0.905966	0.937487
8	0.831107	0.906684	0.938712
9	0.831268	0.905924	0.939220
10	0.831896	0.907037	0.939450
11	0.832290	0.907351	0.938901
12	0.833100	0.908446	0.939202
13	0.833131	0.908638	0.939639
14	0.833108	0.909123	0.940898
15	0.834256	0.908886	0.940724
