# Supplementary material for: Evidential deep learning for trustworthy prediction of enzyme commission number
Source: Brief Bioinform. 2023 Nov 22;25(1):bbad401. doi: 10.1093/bib/bbad401 (PMC10664415; doi:10.1093/bib/bbad401)
Supplement: Supplementary_Table_S2_bbad401 [file supplementary_table_s2_bbad401.pdf]

Table S2. Performance comparison with the newly registered 858 enzymes in Swiss-Prot

Methods	Precision	Recall	F1-score	Predictions	Execution Time
ECPICK	**0.8675**	**0.8845 (0.4196)**	**0.8759 (0.5660)**	**407**	**11s**
ECPred	0.7439	0.7262 (0.1422)	0.7349 (0.2387)	184	7h 24m 59s
DeepEC	0.6993	0.5983 (0.2413)	0.6449 (0.3588)	308	26s
EFICAz2.5	0.5786	0.6322 (0.2704)	0.6042 (0.3685)	294	13h 40m 23s
DETECTv2	0.1998	0.5216 (0.1970)	0.2889 (0.1984)	149	28m 56s

Note: The numbers in parathesis indicates measurements only considering the protein sequence predicted by the models, including protein samples failed to predict.
