# Supplementary material for: Evidential deep learning for trustworthy prediction of enzyme commission number
Source: Brief Bioinform. 2023 Nov 22;25(1):bbad401. doi: 10.1093/bib/bbad401 (PMC10664415; doi:10.1093/bib/bbad401)
Supplement: Supplementary_Table_S3_bbad401 [file supplementary_table_s3_bbad401.pdf]

Table S3. Performance comparison with the protein sequences of three reference genomes in KEGG

	Methods	Precision	Recall	F1-score	Predictions	Execution Time
Bacillus subtilis 168	ECPICK	**0.8894 (0.7682)**	**0.8894 (0.7682)**	**0.8894 (0.7682)**	**913 (Out of 1,057)**	**6s**
	ECPred	0.8174 (0.4459)	0.8131 (0.4447)	0.8153 (0.4453)	578 (Out of 1,057)	10h 12m 17s
	DeepEC	0.8533 (0.6834)	0.8512 (0.6821)	0.8522 (0.6828)	847 (Out of 1,057)	21s
	DETECTv2	0.6717 (0.5052)	0.6717 (0.5052)	0.6717 (0.5052)	795 (Out of 1,057)	14m 18s
	EFICAz2.5	0.7911 (0.6160)	0.7739 (0.6055)	0.7824 (0.6107)	827 (Out of 1,057)	15h 33m 6s
Escherichia coli K-12 MG1655	ECPICK	**0.9042 (0.8535)**	**0.9042 (0.8535)**	**0.9042 (0.8535)**	**1,211 (Out of 1,283)**	**7s**
	ECPred	0.7977 (0.4278)	0.7954 (0.4271)	0.7965 (0.4275)	689 (Out of 1,283)	12h 35m 27s
	DeepEC	0.8508 (0.6768)	0.8492 (0.6758)	0.8500 (0.6763)	1,021 (Out of 1,283)	24s
	DETECTv2	0.6484 (0.4801)	0.6484 (0.4801)	0.6484 (0.4801)	950 (Out of 1,283)	16m 51s
	EFICAz2.5	0.7875 (0.6305)	0.7730 (0.6212)	0.7802 (0.6258)	1,031 (Out of 1,283)	19h 10m
Streptomyces sp. PAMC26508	ECPICK	**0.8351 (0.5544)**	**0.8351 (0.5544)**	**0.8351 (0.5544)**	922 (Out of 1,389)	**7s**
	ECPred	0.7414 (0.3117)	0.7414 (0.3117)	0.7414 (0.3117)	584 (Out of 1,389)	13h 1m 48s
	DeepEC	0.8123 (0.3427)	0.8123 (0.3427)	0.8123 (0.3427)	586 (Out of 1,389)	25s
	DETECTv2	0.6484 (0.4801)	0.6484 (0.4801)	0.6484 (0.4801)	**1,002** (Out of 1,389)	21m
	EFICAz2.5	0.7172 (0.4775)	0.7072 (0.4730)	0.7122 (0.4752)	929 (Out of 1,389)	21h 31m 45s
