# Supplementary material for: Evidential deep learning for trustworthy prediction of enzyme commission number
Source: Brief Bioinform. 2023 Nov 22;25(1):bbad401. doi: 10.1093/bib/bbad401 (PMC10664415; doi:10.1093/bib/bbad401)
Supplement: BB_ECPICK_Supplementary_Final_bbad401 [file bb_ecpick_supplementary_final_bbad401.pdf]

SUPPLEMENTARY

Evidential deep learning for trustworthy prediction of enzyme commission number

So-Ra Han[†1,5], Mingyu Park[†2,5], Sai Kosaraju[3], JeungMin Lee[2,5], Hyun Lee[2,5,6], Jun Hyuck Lee[4], Tae-Jin Oh[*1,5,6,7], and Mingon Kang[*3]

[1] Department of Life Science and Biochemical Engineering, Sun Moon University, Asan, Republic of Korea
[2] Division of Computer Science and Engineering, Sun Moon University, Asan, Republic of Korea
[3] Department of Computer Science, University of Nevada, Las Vegas, NV, USA
[4] Research Unit of Cryogenic Novel Material, Korea Polar Research Institute, Incheon, Republic of Korea
[5] Bio Big Data-based Chungnam Smart Clean Research Leader Training Program, SunMoon University, Asan, Republic of Korea
[6] Genome-based BioIT Convergence Institute, Asan, Republic of Korea
[7] Department of Pharmaceutical Engineering and Biotechnology, Sun Moon University, Asan, Republic of Korea
[†] Authors contributed equally
[*] These authors share corresponding and senior authorship. To whom correspondence should be addressed. Mingon Kang (Tel: +1-702-895-4884; Email: mingon.kang@unlv.edu) and Tae-Jin Oh (Tel: +82-41-530-2677; Email: tjoh3782@sunmoon.ac.kr)

S1. Related Methods

Current computational methods to predict EC numbers are categorized into three categories: (1) protein structure-based, (2) sequence similarity-based, and (3) machine learning-based methods. First, protein structure-based models (e.g., Cofactor[1], i-tasser suite[2]) predict enzyme functions based on protein structure reference databases (e.g., Protein Database Bank, PDB). The protein structure-based approach examines templates of protein structures (or enzyme references), or performs homology analysis with known structures through protein threading[3,4], which can effectively identify the most similar folds and functional sites[5,6]. However, high-quality protein structure databases are still lacking due to high-cost and time-consuming biological experiments[7]. Moreover, EC number predictions of new microorganisms, which in most protein structures are unknown, are inaccurate. Second, the sequence similarity-based approach (e.g., EFICAz[8], ModEnzA[9], PRIAM[10], and EnzML[11,12]) characterizes protein functions based on enzyme references that have high sequence similarities to a query enzyme[13,14]. In this approach, motif sites are identified using multiple sequence alignment, and roles of each amino acid (AA) (e.g., forming binding sites) are assessed[24]. The approach often fails to predict EC numbers accurately when it haves low similarity with references[14]. Moreover, those models require post-hoc analyses, such as multiple sequence alignment, matching domain, and motif site searching, which are computationally expensive[17].

Recently, machine learning-based models have been widely proposed for the EC number prediction. Current state-of-the-art machine learning-based models, including MF-EFP[15], DeepEC[16], DEEPred[17], HECNet[18], HDMLF[19], and CLEAN[20], have significantly improved the predictive performance by automatically recognizing class-specific patterns of protein sequences from a large scale of labelled databases. Especially, machine learning-based models have shown promising performance with unknown enzymes of new species, particularly in microorganisms, compared to protein structured- and sequence similarity-based approaches. However, in spite of the machine learning-based models' success, there is still significant room to improve in terms of predictive performance, model interpretation, and trustworthiness. For instance, one of the most recent deep learning-based models, DeepEC[26], predicts EC numbers up to three levels and four levels separately by using two Convolutional Network Networks (CNN) and comparing the results. Then, homology analysis (e.g., Diamond[21]) is performed if the two results are mismatched. We confirmed around 19% of mismatched results between the two CNN models in the SwissProt and PDB databases, which means DeepEC substantially relies on a sequence similarity approach.

S2. Datasets

We used the three datasets to train the proposed model in this study: (1) Swiss-Prot in Uniprot (https://www.uniprot.org), (2) TrEMBL in Uniprot, and (3) Protein Data Bank (PDB, https: www.rcsb.org) (Table 1). We used the August 2020 version (2020_04 ver) of Swiss-Prot, where totally 563,082 protein sequences of 5,039 EC numbers were available but only 185,962 protein sequences were labeled with four-digit EC numbers. We also used the 2020_04 version of TrEMBL, where 219,174,961 total protein sequences were available, of which we considered

only 21,893,239 labeled with four-digit EC numbers. For PDB, we used 41,162 labeled protein sequences as of October 2020, where the EC numbers were distributed over 3,564 EC classes.

The datasets were mainly used for the ablation study and model training for all the experiments. Swiss-Prot and PDB were labeled their EC numbers with biological experiments, but TrEMBL datasets were computationally predicted, which have not been biologically reviewed. (1) Thus, we used only curated samples in Swiss-Prot and PDB for the ablation study. (2) To make TrEMBL more reliable for the final model, we refined the EC number annotation of the TrEMBL dataset by the strategy that the DeepEC study used[16]. We considered Swiss-Prot and PDB databases as references and re-labeled the TrEMBL dataset by searching the most matched sequences in Swiss-Prot and PDB via DIAMOND. We excluded protein sequences that have sizes lower than 10. Then, the final model was trained with the Swiss-Prot, reannotated TrEMBL, and PDB datasets.

Table 1. Datasets used for this study

	Database	# of sequences (< 20 samples)	# of EC numbers (< 20 samples)
Ablation study	Swiss-Prot	185,962 (7,447)	1,751 (573)
	PDB	34,936 (6,148)	1,007 (491)
Final model	Swiss-Prot + TrEMBL + PDB	23,452,459 (3,823)	4,810 (273)

S3. Ablation studies and performance comparison

We conducted an ablation study to compare the predictive performance with the methods that inspired our model development, including Hierarchical Multi-label Classification Networks (HMCN)[22] and DeepEC[16]. In this experiment, we used the same input data of ECPICK's encoding layer for HMCN for a fair comparison. Comparing to HMCN, ECPICK introduces the flattened latent variables, $\mathbf{v}$, to the hierarchical layers in (1)-(2) and (4) as well as feeding the outcomes in the previous depth to the next depth's classification in (6). We adapted DeepEC's encoding layer for protein sequences. DeepEC contains three CNNs, where CNN1 determines if the protein sequence is an enzyme or not; CNN2 and CNN3 classifies EC numbers up to three and four levels, respectively. If CNN2 and CNN3 are not matched, Diamond is performed. We considered CNN3 of DeepEC only, which is a pure deep learning model, and the final result of DeepEC with Diamond, as benchmark models, in this ablation study.

We used Swiss-Prot and PDB databases for the experiments of the ablation study. We did not consider TrEMBL database due to its potential bias to Swiss-Prot and PDB through the re-annotation process. We randomly split the Swiss-Prot and PDB datasets into 80% and 20% for training and test data by stratified random sampling to preserve the ratio of classes in the training and test data. The training data was split again into 80% and 20% for training and validation data in the same manner. We repeated the experiments five times for reproducibility. For the evaluation, we computed weighted accuracy/macro/micro-averaged F1-scores with top-ranked predictions for multi-label classification. In the experiments, we predicted EC numbers with the highest, top-2, and top-5 posterior probabilities, without thresholding. In the top-1 ranked prediction, we considered a true positive only if the EC number of the highest probability is matched with the ground truth. In the top-2 (or top-5) ranked predictions, ECPICK assigned two (or five) EC numbers of the top-2 (or top-5) highest posterior

probabilities to the given protein sequence, and we considered it a true positive if the ground truth was among the predictions.

ECPICK showed the highest weighted accuracy and macro-/micro-averaged F1-scores in the most settings (Table 2). In Swiss-Prot, ECPICK outperformed the other benchmark models. ECPICK produced weighted accuracy of 77.4±0.5, macro F1-score of 79.1±0.4, and micro F1-score of 88.8±0.2, which improved 11.8%, 13.8%, and 9.9%, respectively, compared to the pure DeepEC without Diamond (CNN3 only in DeepEC). The competitive performance of DeepEC with Diamond substantially resulted from the similarity-based prediction using Diamond due to the mismatch between CNN2 and CNN3 in DeepEC. Around 18% of the predictions between CNN2 and CNN3 were mismatched. DeepEC with Diamond did not continuously improve the performance on the Top-5 predictions due to its dependence on Diamond. Whereas ECPICK constantly improved the prediction performance in the Top-2 and Top-5 predictions. ECPICK showed performance improvements of 15.6%, 13.7%, and 8% on the weighted accuracy and macro/micro F1-scores respectively, compared to DeepEC with Diamond in the top 5 predictions. HMCN showed better performance than DeepEC CNN3 in the most experiments, which indicates a hierarchical classifier is a better choice for the EC number prediction. ECPICK also showed higher macro-averaged F1-scores on the EC number samples where less than 20 samples were available in the training data, mainly due to the advantage of the hierarchical layers. DeepEC with Diamond gave higher performance with top-1 prediction in PDB, because Diamond mainly improved the performance on PDB that mostly contains enzymes whose structures are known.

Table 2. Ablation study

	**Methods**	Top-1			Top-2			Top-5			Small Class (MA. F1)		
		W. Acc.	MA.F1	MI. F1	W. Acc.	MA.F1	MI. F1	W. Acc.	MA.F1	MI. F1	Top 1	Top 2	Top 5
Swiss-Prot	HMCN	.697 ±.013	.729 ±.011	.860 ±.004	.787 ±.012	.820 ±.010	.928 ±.004	.833 ±.010	.865 ±.008	.950 ±.002	.590 ±.019	.693 ±.022	.760 ±.017
	DeepEC CNN3	.692 ±.002	.695 ±.001	.808 ±.002	.784 ±.002	.793 ±.002	.885 ±.001	.838 ±.003	.849 ±.002	.924 ±.001	.594 ±.003	.703 ±.005	.773 ±.007
	DeepEC+Diamond	.760 ±.004	.787 ±.003	.882 ±.001	.794 ±.003	.822 ±.003	.904 ±.001	.794 ±.003	.822 ±.002	.903 ±.001	**.700 ±.007**	.747 ±.005	.754 ±.004
	ECPICK	**.774 ±.005**	**.791 ±.004**	**.888 ±.002**	**.872 ±.003**	**.891 ±.002**	**.959 ±.008**	**.918 ±.002**	**.935 ±.002**	**.976 ±.001**	.686 ±.006	**.811 ±.002**	**.883 ±.002**
PDB	HMCN	.529 ±.020	.566 ±.017	.537 ±.010	.651 ±.023	.700 ±.020	.671 ±.011	.719 ±.019	.770 ±.016	.819 ±.010	.537 ±.022	.664 ±.024	.726 ±.018
	DeepEC CNN3	.555 ±.005	.503 ±.004	.435 ±.004	.654 ±.004	.611 ±.005	.541 ±.005	.700 ±.004	.663 ±.004	.639 ±.005	.515 ±.004	.617 ±.004	.660 ±.003
	DeepEC+Diamond	**.643 ±.005**	**.664 ±.003**	**.607 ±.002**	.676 ±.006	.700 ±.005	.640 ±.002	.662 ±.026	.693 ±.007	.654 ±.025	**.656 ±.006**	.688 ±.005	.678 ±.018
	ECPICK	.621 ±.007	.638 ±.006	.581 ±.005	**.744 ±.006**	**.778 ±.004**	**.718 ±.003**	**.804 ±.007**	**.839 ±.006**	**.861 ±.005**	.623 ±.008	**.752 ±.007**	**.806 ±.008**

S4. Tuning hyper-parameters of ECPICK

ECPICK involves three non-trainable hyper-parameters: (1) learning rate, (2) dropout ratio, (3) weight (β) between global and local flow layers in the output layers. We empirically obtained the optimal hyper-parameters through the experiment using Swiss-Prot, where we computed loss and weighted accuracy with varying hyper-parameter values on the split data into training

and validation. The overfitting issue is solved by dropout and the ensemble strategy. The model bias to the majority classes (due to the data imbalance) was tackled by class weights when training the model. The optimal hyper-parameters were 0.001, 0.8, and 0.6 for the learning rate, dropout ratio, and β, respectively. We used adam optimizers (beta: 0.9 ~ 0.999, eps: 1e-7).

S5. Thresholding with false discovery rates for multiple reliable EC number predictions

ECPICK produces a posterior probability for each EC number class, $\mathrm{Pr}(\mathrm{C_i}|\mathbf{x}), 1 \leq i \leq \mathrm{ECs}$ for the multi-label classification problem, where $\mathrm{C_i}$ is a label of the ith four-digit EC number, ECs is the total number of available EC numbers, and $\mathbf{x}$ is a protein sequence. Then, the final EC number prediction is determined with the threshold (θ) as:

$$\mathrm{Pr}(\mathrm{C_i}|\mathbf{x}) > \theta. \quad (12)$$

We obtained the optimal threshold (θ) with False Discovery Rates (FDR). False Discovery Rates (FDR) analysis was conducted to measure the expected proportion of incorrectly EC assignments by using only non-enzyme protein sequences. We obtained the non-enzyme protein sequences (N=22,168) from DEEPre[23]. The thresholds that allow 5% and 1% FDR errors were 0.19 and 0.6, respectively (Fig. S1). In this study, we used the threshold of FDR = 0.05, $\theta = 0.19$, for all this paper's experiments. ECPICK does not require to classify whether the given protein sequence is an enzyme, as a separate pre-processing process, since the thresholding in the final decision determines non-enzymes.

S6. Computing importance scores for trustworthy predictions and identifying potential motif sites

ECPICK identifies discriminative AAs on the given protein sequences that mainly contribute to the prediction, which may correspond to well-known or potential motif sites or conserved regions of the protein. To identify discriminative AAs, we compute an importance score on each AA. An importance score (τ) on an AA of a given protein sequence, τ_i $(1 \leq i \leq 1{,}000)$, is computed based on top-K activation maps in the convolutional layers of the optimal ECPICK model (K=50 in this study). Importance scores are calculated on each of three convolutional tracks with the kernel sizes of 4×21, 8×21, and 16×21, respectively, and the importance scores of the three tracks are summed up. Then, the importance scores of the ten models in the ensemble learning are averaged for the final importance scores.

Let ϕ_i^k $(1 < k < K)$ be the activation value of the ith AA in the kth activation map. To calculate the importance score (τ_i) on the i^{th}AA, we rank the 128 activation maps in the convolutional layer of each convolutional track by computing the activation map scores (α_m, $1 \leq m \leq 128$), where m is an index of the activation maps. Note that ECPICK has only one convolutional layer in each convolutional track. An activation map score is calculated by the partial derivative of the EC number prediction (y^c) with respect to a max-pooling node (β_m, 1 < m < 128):

$$\alpha_\mathrm{m} = \frac{\partial y^c}{\partial \beta_m}. \quad (13)$$

Once the activation maps are ranked by α_m, we consider top-K activations maps, which are discriminative to the classification. Each top-K activation map consists of $n = 1{,}000 - \ell + 1$

activation node values (ϕ), where ℓ ($\ell = 4, 8, 16$) is the length of the kernel. Then, we calculate activation scores on the AA subsequence of length ℓ ($\boldsymbol{\zeta^{\ell}}$; $l = 4, 8, 16$) for each convolutional track by summing the activation values of top-*K* activations maps for each AA, as shown in (14).

$$\boldsymbol{\zeta^{\ell}} = \begin{bmatrix} \zeta_1^{\ell} \\ \vdots \\ \zeta_n^{\ell} \end{bmatrix} = \begin{bmatrix} \phi_1^1 + \phi_2^1 + & \cdots & +\phi_{\{K-1\}}^1 + \phi_K^1 \\ \vdots & \ddots & \vdots \\ \phi_1^n + \phi_2^n + & \cdots & +\phi_{\{K-1\}}^n + \phi_K^n \end{bmatrix} \tag{14}$$

where n is the length of activation map, ($n = 1000 - \ell + 1$). To calculate the importance score (τ_i^l) of the i^{th}AA for the CNN track with kernel size l, we assumed values $\zeta_{n+1}^{\boldsymbol{\ell}}, .., \zeta_{1000}^{\boldsymbol{\ell}}$ as zeros to align $\boldsymbol{\zeta^{\ell}}$ with the length of the AA sequence (EC Pick architecture used CNNs with stride length 1, and no padding allowed). Then, we take average of l consecutive $\boldsymbol{\zeta_i^{\ell}}$ values as shown in equation:

$$\tau_i^{\boldsymbol{\ell}} = \frac{1}{l}\left(\sum_{j=1}^{\min(\mathrm{i}, \boldsymbol{\ell})} \zeta_{i-j+1}^{\boldsymbol{\ell}}\right). \tag{15}$$

The $\tau^{\boldsymbol{\ell}}$ value computing in (3) is an importance score for the protein sequence on track with kernel size l. Then the final importance score τ is calculated using: $\tau = \tau^4 + \tau^8 + \tau^{16}$. In ECPICK, we used ensemble learning. The final τ is calculated by the summation of individual τ of all the models as:$\tau = \sum_{\{j=1\}}^{j} \tau_j$.

S7. CAZyme Functional Screening

In this study, we validated enzymatic activity through azurine cross-linked (AZCL) analysis, which hinges on the observable solubilization of minute AZCL polysaccharide particles as a surrogate for evaluating CAZyme functionality. The AZCL assay reveals that the formation of a blue halo on solid media serves as a clear indicator of polysaccharide-degrading activity in the presence of enzymatic activity and offers robust validation of CAZyme functionality[24]. To rapidly detect CAZyme, strain PAMC26508 was cultivated in R2A medium (BD Difco, USA). The AZCL active medium consisted of 2% agarose with xanthan gum 0.1g, and each polysaccharide substrate from 25 mM sodium phosphate buffer 100mL at pH 5.5. For each assay, 20 µL of sonicated cell culture were dispensed onto AZCL plates. Incubation was carried out at 15℃ for a duration spanning from 7 to 10 days. The formation of a discernible blue halo was meticulously documented as confirmation of enzymatic activity. The AZCL-polysaccharide substrate utilized in these experiments was procured from Megazyme (Ireland, https://www.megazyme.com/).

REFERENCE

1. Zhang, C., Freddolino, P. L. & Zhang, Y. COFACTOR: improved protein function prediction by combining structure, sequence and protein--protein interaction information. *Nucleic Acids Res.* **45**, W291--W299 (2017).

2. Yang, J. *et al.* The I-TASSER Suite: protein structure and function prediction. *Nat. Methods* **12**, 7–8 (2015).

3. Roy, A., Yang, J. & Zhang, Y. COFACTOR: an accurate comparative algorithm for

structure-based protein function annotation. *Nucleic Acids Res.* **40**, W471--W477 (2012).

4. Peng, J. & Xu, J. A multiple-template approach to protein threading. *Proteins Struct. Funct. Bioinforma.* **79**, 1930–1939 (2011).

5. Amidi, A., Amidi, S., Vlachakis, D., Paragios, N. & Zacharaki, E. I. A machine learning methodology for enzyme functional classification combining structural and protein sequence descriptors. *Lect. Notes Comput. Sci. (including Subser. Lect. Notes Artif. Intell. Lect. Notes Bioinformatics)* **9656**, 728–738 (2016).

6. Heckmann, D. *et al.* Machine learning applied to enzyme turnover numbers reveals protein structural correlates and improves metabolic models. *Nat. Commun.* **9**, (2018).

7. Li, Y. *et al.* DEEPre: Sequence-based enzyme EC number prediction by deep learning. *Bioinformatics* **34**, 760–769 (2018).

8. Tian, W., Arakaki, A. K. & Skolnick, J. EFICAz: a comprehensive approach for accurate genome-scale enzyme function inference. *Nucleic Acids Res.* **32**, 6226–6239 (2004).

9. Desai, D. K., Nandi, S., Srivastava, P. K. & Lynn, A. M. ModEnzA: accurate identification of metabolic enzymes using function specific profile HMMs with optimised discrimination threshold and modified emission probabilities. *Adv. Bioinformatics* **2011**, (2011).

10. Claudel-Renard, C., Chevalet, C., Faraut, T. & Kahn, D. Enzyme-specific profiles for genome annotation: PRIAM. *Nucleic Acids Res.* **31**, 6633–6639 (2003).

11. Ferrari, L. De, Aitken, S., Hemert, J. Van & Goryanin, I. Multi-label prediction of enzyme classes using InterPro signatures. *Mach. Learn. Syst. Biol.* 1–20 (2010).

12. De Ferrari, L., Aitken, S., van Hemert, J. & Goryanin, I. EnzML: Multi-label prediction of enzyme classes using InterPro signatures. *BMC Bioinformatics* **13**, (2012).

13. Arakaki, A. K., Huang, Y. & Skolnick, J. EFICAz 2: enzyme function inference by a combined approach enhanced by machine learning. *BMC Bioinformatics* **10**, 1–15 (2009).

14. Quester, S. & Schomburg, D. EnzymeDetector: an integrated enzyme function prediction tool and database. *BMC Bioinformatics* **12**, 1–13 (2011).

15. Xiao, X. *et al.* MF-EFP: Predicting Multi-Functional Enzymes Function Using Improved Hybrid Multi-Label Classifier. *IEEE Access* **8**, 50276–50284 (2020).

16. Ryu, J. Y., Kim, H. U. & Lee, S. Y. Deep learning enables high-quality and high-throughput prediction of enzyme commission numbers. *Proc. Natl. Acad. Sci. U. S. A.* (2019) doi:10.1073/pnas.1821905116.

17. Rifaioglu, A. S., Do\ugan, T., Martin, M. J., Cetin-Atalay, R. & Atalay, V. DEEPred: automated protein function prediction with multi-task feed-forward deep neural networks. *Sci. Rep.* **9**, 1–16 (2019).

18. Memon, S. A., Khan, K. A. & Naveed, H. HECNet: A hierarchical approach to enzyme function classification using a siamese triplet network. *Bioinformatics* **36**, (2020).

19. Shi, Z. *et al.* Enzyme Commission Number Prediction and Benchmarking with Hierarchical Dual-core Multitask Learning Framework. *Research* **6**, (2023).

20. Yu, T. *et al.* Enzyme function prediction using contrastive learning. *Science (80-.).* **379**, (2023).

21. Buchfink, B., Xie, C. & Huson, D. H. Fast and sensitive protein alignment using DIAMOND. *Nat. Methods* **12**, 59–60 (2015).

22. Wehrmann, J., Cerri, R. & Barros, R. C. Hierarchical multi-label classification networks. in *35th International Conference on Machine Learning, ICML 2018* vol. 12 (2018).

23. Li, Y. *et al.* DEEPre: sequence-based enzyme EC number prediction by deep learning. *Bioinformatics* **34**, 760–769 (2018).

24. Kračun, S. K. *et al.* A new generation of versatile chromogenic substrates for high-throughput analysis of biomass-degrading enzymes. *Biotechnol. Biofuels* **8**, (2015).
